# Ultrasonographic findings in a cow with vascular hamartoma of the liver: case report

**DOI:** 10.1186/1746-6148-7-52

**Published:** 2011-09-04

**Authors:** Ueli Braun, Luzia Trösch, Christian Gerspach, Katrin Brosinski, Monika Hilbe

**Affiliations:** 1 Department of Farm Animals, Vetsuisse Faculty, University of Zurich, Winterthurerstrasse 260, 8057 Zurich, Switzerland; 2 Institute of Veterinary Pathology, Vetsuisse Faculty, University of Zurich, Winterthurerstrasse 260, 8057 Zurich, Switzerland

## Abstract

**Background:**

This is the first description of the ultrasonographic findings in a cow with vascular hamartoma of the liver.

**Case presentation:**

Ultrasonographic examination of a six-year-old Swiss Braunvieh cow revealed an excessive number of hypoechogenic blood vessels in the liver parenchyma and a thrombus in the right hepatic vein. The activities of the liver enzymes and the concentration of bilirubin were within the reference ranges. At postmortem examination, a poorly delineated, non-encapsulated lesion, measuring approximately 10 cm × 10 cm in diameter, was found in the right liver lobe. The cut surface of the lesion was sponge-like and contained extremely dilated blood vessels, one of which was occluded with a branching red thrombus. A vascular hamartoma of the liver with thrombosis was diagnosed based on the histological findings.

**Conclusions:**

To our knowledge, this is the first description of the ultrasonographic findings of vascular hamartoma of the liver in a cow. Hamartoma should be considered part of the differential diagnosis in cows with an abnormally large number of blood vessels in the liver parenchyma. This case report broadens the spectrum of liver diseases and ultrasonographic findings of the liver in cattle.

## Background

The term hamartoma refers to focal disordered overgrowth of mature tissue that is indigenous to the organ involved [1]. Vascular hamartomas may occur at any site in the body. Most hamartomas are present at birth and their growth is coordinated with that of the surrounding tissue. Mesenchymal (largely vascular) hamartomas of the human liver, though rare, are a distinct clinicopathological entity, occurring primarily in infants and young children and seldom in adults [2-6]. Hamartomas in cattle have also been described primarily in calves, young animals and rarely in adults. In calves, hamartoma of the bile ducts [7], lungs [8], vagina [9], testicle [10], smooth muscle of the abomasum [11] and gingiva [12-15] has been reported. In adult cattle, hamartoma of the liver [16], ovary [17] and heart [18] has been described. Hamartoma of the liver in human fetuses and children manifests clinically as abdominal enlargement. However, in cattle, hamartoma has not been diagnosed *in vivo *and is usually an incidental finding at necropsy or slaughter [16]. The goal of this case report was to describe the ultrasonographic findings in a six-year-old Swiss Braunvieh cow with hamartoma of the liver.

## Case presentation

**Figure 1:**
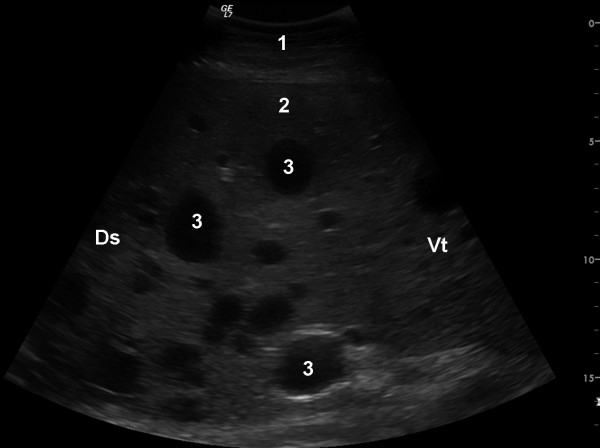
**Ultrasonogram of vascular hamartoma of the liver**. Ultrasonogram of the liver of a six-year-old Swiss Braunvieh cow showing many dilated liver veins within a vascular hamartoma. The image was obtained from the 11th intercostal space using a 5.0 MHz convex transducer. 1 Lateral abdominal wall, 2 Liver, 3 Dilated liver veins, Ds Dorsal, Vt Ventral.

**Figure 2:**
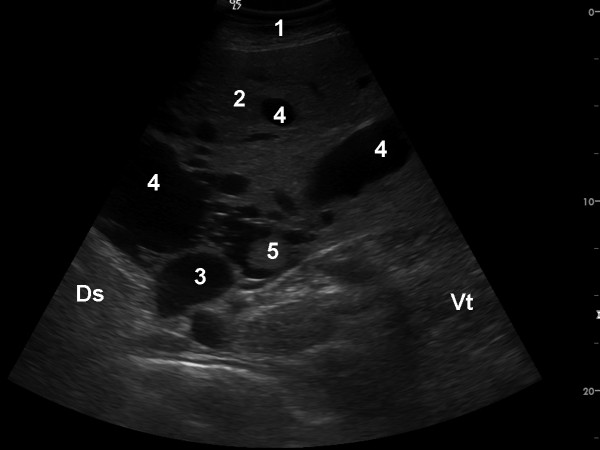
**Thrombus in a liver vein in a cow with vascular hamartoma of the liver**. Ultrasonogram of the liver of a six-year-old Swiss Braunvieh cow with a vascular hamartoma of the liver. Many dilated liver veins and an echogenic thrombus, in what is likely the right hepatic vein, are evident. The image was obtained from the 11th intercostal space using a 5.0 MHz convex transducer. 1 Lateral abdominal wall, 2 Liver, 3 Caudal vena cava, 4 Dilated liver veins, 5 Echogenic thrombus, Ds Dorsal, Vt Ventral.

**Figure 3:**
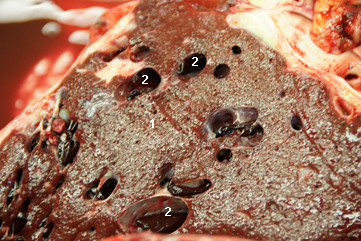
**Dilated liver veins in a cow with vascular hamartoma**. Dilated liver veins are apparent in a six-year-old Swiss Braunvieh cow with a vascular hamartoma of the liver. 1 Liver, 2 Dilated liver veins.

**Figure 4:**
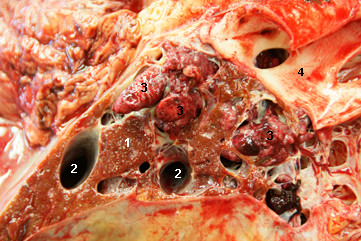
**Postmortem findings in a cow with vascular hamartoma of the liver**. Multiple red thrombi in a six-year-old Swiss Braunvieh cow with a vascular hamartoma of the liver. 1 Liver, 2 Dilated liver veins, 3 Red thrombi, 4 Caudal vena cava.

**Figure 5:**
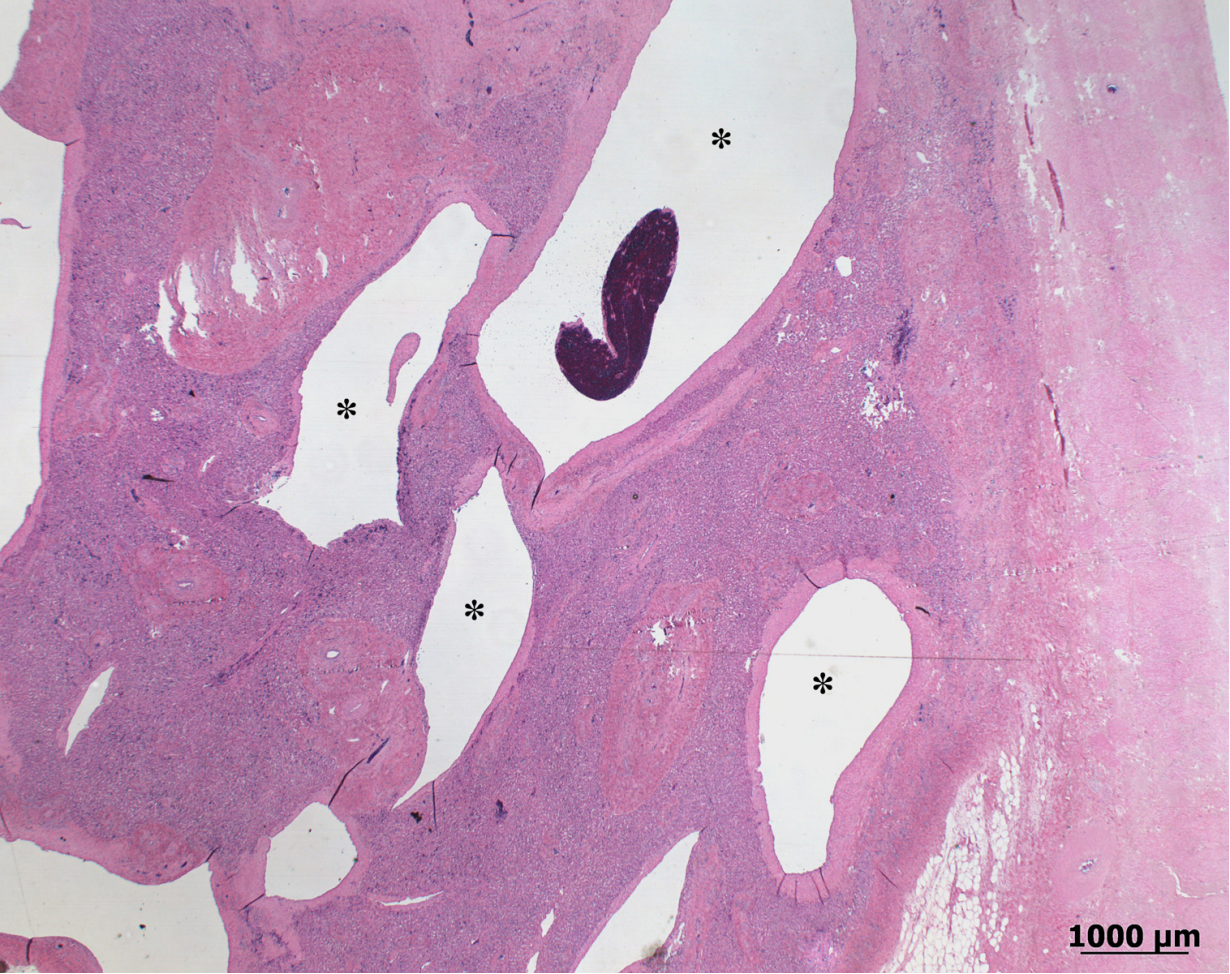
**Histology of the hamartoma in the right hepatic lobe**. A poorly delineated, non-encapsulated, cavernous mass composed of well-differentiated arterial and venous spaces that are lined by endothelium (asterisk). The spaces vary in size and are separated from each other by well-differentiated connective tissue, which has replaced the hepatic parenchyma. (H&E, Bar 1000 μm).

**Figure 6:**
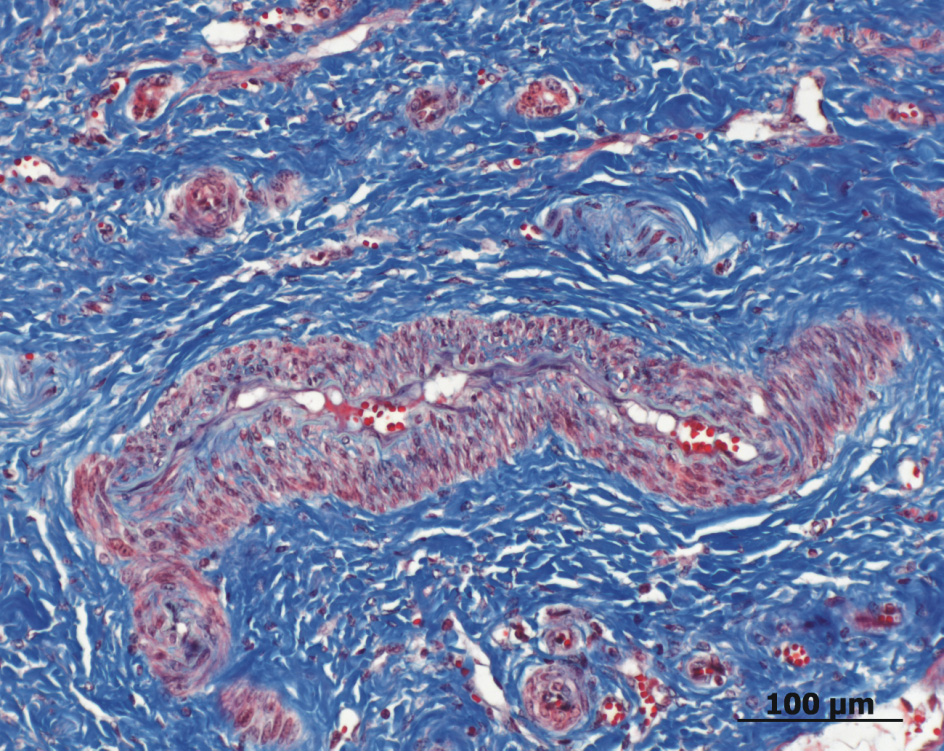
**Histology of the connective tissue component of the vascular hamartoma**. The fibrous tissue is well differentiated and arranged in bundles of spindle-shaped cells (blue). The connective tissue is interspersed with bile duct proliferation. The lamina muscularis of the arterial spaces is hypertrophied (Gomori's Blue Trichrome stain, Bar 100 μm).

**Figure 7:**
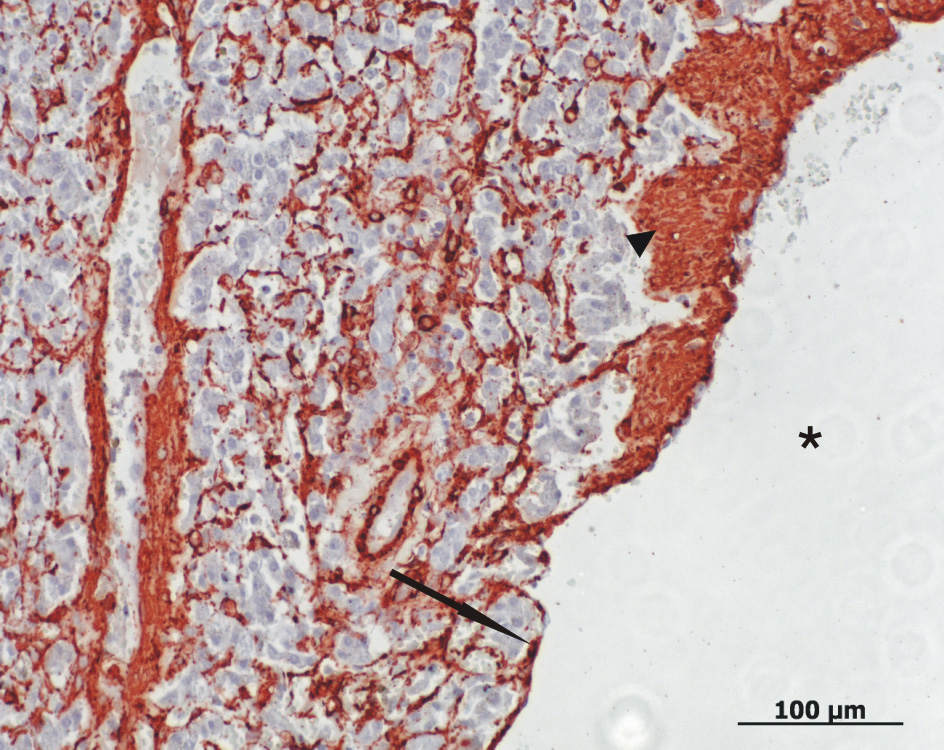
**Histology of a dilated vessel in the vascular hamartoma**. The lamina muscularis of the dilated vessel (asterisk) has areas of atrophy (arrow) as well as hypertrophy (arrowhead). (Smooth muscle α-actin, Bar 100 μm).

The cow was referred to our clinic because of regurgitation, which will not be discussed in this report. Abdominal ultrasonography using a 5.0 MHz-convex transducer revealed an abnormally large number of hypoechogenic blood vessels in the liver parenchyma (Figure 1) and a thrombus in a vein, which was thought to be the right hepatic vein (Figure 2). The caudal vena cava had a normal triangular shape in cross section, and the portal vein had typical stellate ramifications. Neither of these two vessels were dilated, and the liver appeared normal in size. Abdominal effusion was not seen. The activities of gammaglutamyl transferase (γ-GT), aspartate aminotransferase (AST), glutamate dehydrogenase (GLDH) and sorbitol dehydrogenase (SDH) and the concentration of bilirubin were within the reference ranges of the Clinical Laboratory, Vetsuisse Faculty, University of Zurich (γ-GT 25 U/l, reference range 13 - 32 U/l; AST 40 U/l, reference range 30 - 103 U/l; GLDH 13.3 U/l, reference range 4 - 18 U/l; SDH 5.2 U/l, reference range 4.0 - 7.4; bilirubin 4.2 μmol/l, reference range 1.5 - 6.5 μmol/l). The cow was euthanased because of regurgitation. Postmortem examination revealed a perioesophageal abscess, which was likely the cause of regurgitation, moderate ascites and two abomasal ulcers. The right hepatic lobe had a cavernous, poorly delineated, non-encapsulated mass measuring 10 cm in diameter. The cut surface of the lesion was sponge-like (Figure 3) and contained extremely dilated blood vessels, one of which was occluded with a branching red thrombus (Figure 4). Blood vessels in the remainder of the liver were also markedly dilated. The gallbladder contained numerous *Dicrocoelium dendriticum *flukes. Histological examination of H&E-stained sections of the right liver lobe revealed that the parenchyma was obliterated by a non-encapsulated, cavernous lesion consisting of well-differentiated, blood-filled venous and arterial vascular spaces. The endothelial cells were immunohistochemically positive for factor VIII related antigen. These spaces were separated by a well-differentiated connective tissue arranged in tightly-packed bundles of spindle-shaped cells, which varied in width and were irregularly arranged (Figure 5, 6). In some of the dilated vessels, the lamina muscularis had areas of atrophy as well as hypertrophy (Figure 7). A well-organised red thrombus was found in one of the blood vessels. Focal haemorrhages, regenerative nodules with partially degenerated hepatocytes, accumulation of degenerated neutrophils and marked proliferation of bile ducts were seen at the margin of the lesion. Elsewhere in the liver, there was severe portal fibrosis and bile duct proliferation attributable to infestation with *Dicrocoelium dendriticum*. Based on the histological findings, vascular hamartoma of the liver with thrombosis was diagnosed.

Hamartoma of the liver is rare in mammals. In cattle, there is only one report, which described the postmortem diagnosis of liver hamartoma in eight healthy slaughter cattle [16]. Liver hamartoma has been reported in an equine fetus [19] and three dogs [20-22]. In contrast to human beings, liver hamartoma in cattle does not appear to be clinically significant. The bovine cases described were incidental findings during post-slaughter inspection [16]. In the present case report, the liver hamartoma was also a coincidental finding during work-up of a regurgitation problem, in which routine ultrasonography revealed a liver lesion. The ultrasonographic appearance of the lesion was confusing because it did not resemble anything we had seen in the past 20 years. However, the findings were similar to multicystic lesions described for liver hamartoma in human medicine [2,4-6]. In our case, it was not clear initially whether the dilated vessels were blood vessels or bile ducts. The differential diagnosis included dilatation of hepatic vasculature attributable to right-sided cardiac insufficiency or thrombosis of the caudal vena cava; cholestasis and neoplasia were other possible rule-outs. Right ventricular heart failure was ruled out because the cow had a normal heart rate and no jugular distension. The venous thrombus suggested thrombosis of the caudal vena cava because septic thrombi have been described in the caudal vena cava [23] as well as in the hepatic veins [24]. Thrombosis of the caudal vena cava was ruled out because in our experience, this vein is oval to circular in cross-section when dilated [25]. In addition, the pattern of multiple dilated vessels had not been seen previously in cases with thrombosis of the caudal vena cava. The red thrombus found in our patient was likely attributable to poor venous blood flow or blood stasis. Its red colour was due to a high proportion of erythrocytes and relatively few platelets [26]. Similar thrombi were found in four of eight hamartomas in cattle [16]. The ascites seen in our patient was due to portal hypertension [21]. The ultrasonographic findings of the liver were uncharacteristic of cholestasis [25] or liver tumours [27]. The activities of liver enzymes and concentration of bilirubin were also normal, which did not support a diagnosis of cholestasis or liver tumour. A biopsy may have provided an in-vivo diagnosis, but euthanasia was elected for other reasons. However, it is questionable whether biopsy is indicated in a suspected vascular tumor. The findings of this case report provide important reference data for the diagnosis of liver hamartoma in cattle.

## Conclusion

In cows with a greater than normal number of hypoechogenic blood vessels in the liver parenchyma, the differential diagnosis should include vascular hamartoma. To our knowledge, this is the first description of the ultrasonographic findings of vascular hamartoma of the liver in cattle. Vascular hamartoma of the liver must be part of the diffferential diagnosis in cows with dilated liver vessels, normal activities of liver enzymes and a normal concentration of bilirubin. The case reported broadens the spectrum of liver diseases and the ultrasonographic findings of the liver in cattle.

## Consent

Consent was obtained from the owner of the cow for publication of this case report and any accompanying images.

## Authors' contributions

UB prepared the manuscript and supervised the clinical examination, LT and CG examined the cow and performed the ultrasonographic examination, KB and MH performed the postmortem examination. All authors have read and approved the manuscript.
